# Detection of masses in mammograms using a one-stage object detector based on a deep convolutional neural network

**DOI:** 10.1371/journal.pone.0203355

**Published:** 2018-09-18

**Authors:** Hwejin Jung, Bumsoo Kim, Inyeop Lee, Minhwan Yoo, Junhyun Lee, Sooyoun Ham, Okhee Woo, Jaewoo Kang

**Affiliations:** 1 Department of Computer Science and Engineering, Korea University, Seoul, Republic of Korea; 2 Interdisciplinary Graduate Program in Bioinformatics, Korea University, Seoul, Republic of Korea; 3 Department of Radiology, Kangbuk Samsung Medical Center, Seoul, Republic of Korea; 4 Department of Radiology, Korea University Guro Hospital, Seoul, Republic of Korea; Universitatsmedizin Greifswald, GERMANY

## Abstract

Several computer aided diagnosis (CAD) systems have been developed for mammography. They are widely used in certain countries such as the U.S. where mammography studies are conducted more frequently; however, they are not yet globally employed for clinical use due to their inconsistent performance, which can be attributed to their reliance on hand-crafted features. It is difficult to use hand-crafted features for mammogram images that vary due to factors such as the breast density of patients and differences in imaging devices. To address these problems, several studies have leveraged a deep convolutional neural network that does not require hand-crafted features. Among the recent object detectors, RetinaNet is particularly promising as it is a simpler one-stage object detector that is fast and efficient while achieving state-of-the-art performance. RetinaNet has been proven to perform conventional object detection tasks but has not been tested on detecting masses in mammograms. Thus, we propose a mass detection model based on RetinaNet. To validate its performance in diverse use cases, we construct several experimental setups using the public dataset INbreast and the in-house dataset GURO. In addition to training and testing on the same dataset (i.e., training and testing on INbreast), we evaluate our mass detection model in setups using additional training data (i.e., training on INbreast + GURO and testing on INbreast). We also evaluate our model in setups using pre-trained weights (i.e., using weights pre-trained on GURO, training and testing on INbreast). In all the experiments, our mass detection model achieves comparable or better performance than more complex state-of-the-art models including the two-stage object detector. Also, the results show that using the weights pre-trained on datasets achieves similar performance as directly using datasets in the training phase. Therefore, we make our mass detection model’s weights pre-trained on both GURO and INbreast publicly available. We expect that researchers who train RetinaNet on their in-house dataset for the mass detection task can use our pre-trained weights to leverage the features extracted from the datasets.

## Introduction

Abnormalities such as masses, micro-calcifications, and areas of asymmetry and distortion within the breast may indicate the existence of breast cancer. Among these abnormalities, masses are the most representative and common lesion type. However, masses can be easily hidden by overlapping breast tissues, making it difficult to detect them. Moreover, some breast tissues are morphologically similar to masses, and thus misidentified as masses. An undetected mass is a false negative, which delays a patient’s diagnosis until the next screening. A misidentified mass is a false positive, which leads to additional tests including re-screening and biopsy, causing unnecessary anxiety and pain to patients. These problems limit the effectiveness and utility of mammography.

Several computer aided diagnosis (CAD) systems have been developed as a decision support tool for detecting masses. However, CAD systems are only slightly effective in reducing the number of false positives and false negatives, and thus have limited impact on improving diagnosis accuracy [1–3]. This is mainly due to the technical limitations of CAD systems. Before the advent of deep learning, the dominant methods for detecting masses involved extracting pre-defined mass features using image filters [4–8]. Traditional machine learning models with manually crafted features were employed as classifiers [9–16]. However, manual feature extraction and selection are extremely time consuming. Moreover, mammogram variations in density, brightness, contrast, texture, and tissue context make it difficult to define meaningful features.

A convolutional neural networks (CNNs) consists of a number of convolutional layers which can extract features that represent the various contexts of images without feature engineering. Due to this advantage, CNN has become the most widely used method for image interpretation tasks in many domains. After the success of CNNs in standard object detection tasks [17], several studies have exploited the advantages of deep CNNs to overcome the drawbacks of conventional mass detection models. Becker et al. evaluated the diagnostic accuracy of artificial neural networks for the detection of breast cancer and showed that deep learning models can achieve similar accuracy as radiologists [18]. Kooi et al. found mass candidates using a random forest (RF) classifier based on manually designed features, and classified the candidates using a CNN [19]. Dhungel et al. used multi-scale deep belief networks (m-DBNs) and a Gaussian mixture model (GMM) to find mass candidates. Also, a CNN and a random forest (RF) classifier were used to refine the results from numerous candidates [20]. Furthermore, Dhungel et al. improved their previous work by applying more precisely aligned bounding boxes [21]. Akselrod-Ballin et al. employed a region-based CNN [22] and improved the mass detection performance on an in-house dataset [23]. Similarly, Ribli et al. proposed a lesion detection model that employs a region-based CNN [24]. Choukroun et al. proposed a deep learning based model that can be trained without location labels, and perform lesion detection and classification [25]. While most of the mass detection models use CNN as a component, we use an end-to-end object detector based on CNN for the mass detection task.

There are two main object detector types: two-stage object detector and one-stage object detector. The more widely used type is the two-stage object detector. The Region-based Convolutional Neural Network (R-CNN) [26] is a representative two-stage object detector and it drastically improved detection performance. Modifications were made to its network structure to develop the subsequent models Fast R-CNN [27] and Faster R-CNN [28]. The Faster R-CNN model was employed in the study of Akselrod-Ballin et al. [23, 23] and Ribli et al. [24]. However, the main shortcoming of the two-stage object detector is that its complex network architecture makes training and inference less efficient.

The other detector type is the one-stage object detector. One-stage object detectors whose architecture is simpler than that of two-stage object detectors were introduced as an alternative. OverFeat [29], SSD [30], and YOLO [31], all of which are one-stage detectors, have attracted attention due to their fast processing, but they are limited in accuracy. However, RetinaNet [32], a recently proposed one-stage object detector, achieves high performance using Focal Loss function which addresses the drawback of the traditional cross-entropy loss function, while keeping the processing efficient, which is the main advantage of one-stage object detectors. For the mass detection task, we propose a model based on RetinaNet which is a robust region-based deep learning object detector.

Our mass detection model is evaluated on the public mammogram dataset INbreast [33] and the in-house hospital mammogram dataset GURO from Korea University Guro Hospital. Our mass detection model achieves performance comparable to that of the state-of-the-art models in various experimental setups. In addition, we show that the overall performance is maintained or slightly improves when using the combined training data even though the data are obtained from different sources. This demonstrates that performance improvement can be achieved by cross-dataset training where different data including public domain data and a large amount of privately owned data from various sources are used.

The contributions of our work are three-fold:

- We introduce a new mass detection model based on RetinaNet which is a state-of-the-art one-stage object detector that use a convolutional neural network.
- Through an experimental evaluation, we show that our model effectively extracts invariant mass features from the single dataset as well as the combined dataset whose mammograms are collected from different sources, which suggests that our model can be applied to different patient groups.
- We make our weights pre-trained on both the public dataset and the in-house dataset publicly available so that other researchers and practitioners in the community can easily build high performance mass detection models that can be further trained on their in-house datasets using our pre-trained weights.

The rest of the paper is organized as follows. In the Methods section, we describe our one-stage mass detection model. We explain the results of our experimental evaluation in the Experiments and Results section. We discuss the limitations of our proposed model and future direction in the Discussion section, and we conclude the paper in the conclusion section.

## Methods

### Ethics statement

The use of in-house mammograms in this study was approved by a Institutional Review Board (IRB) of Korea University Guro Hospital (approval number: KUGH15342-006).

### Model description

**RetinaNet**: RetinaNet [32] is a one-stage object detector presented at the 2017 International Conference on Computer Vision (ICCV) by FAIR (Facebook AI Research). The author of RetinaNet identified class imbalance as the most critical reason why the performance of one-stage detectors lags behind that of two-stage detectors. To improve performance, RetinaNet employs a simple but effective novel loss function called Focal Loss which allows it to focus more on difficult samples. Using a one-stage network architecture with Focal Loss, RetinaNet achieves state-of-the-art performance in terms of accuracy and running time.

**Fig 1:**
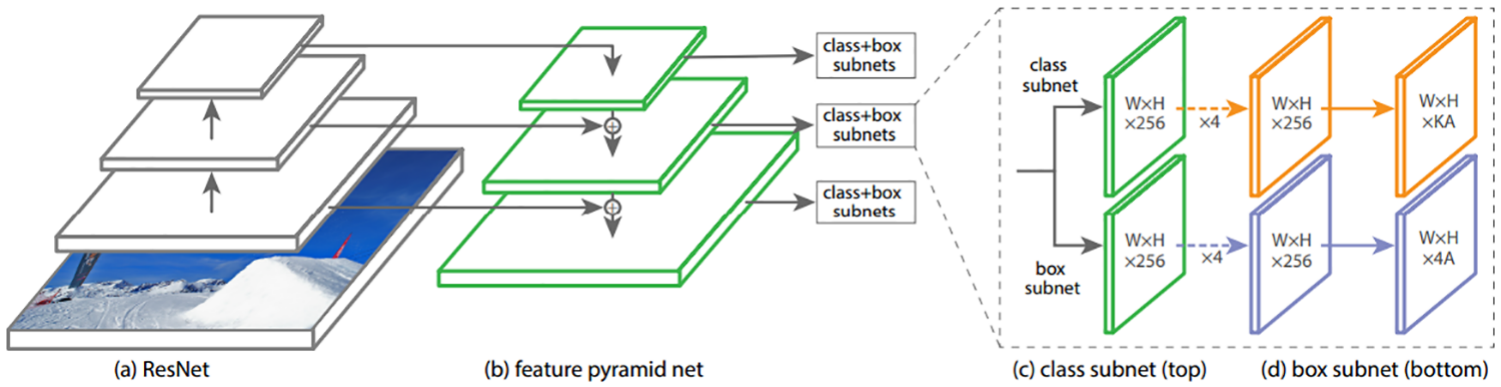
The network architecture of RetinaNet. RetinaNet uses the Feature Pyramid Network (FPN) [34] on top of the convolutional neural network ResNet [35] as a backbone network (a) to generate a rich convolutional feature pyramid (b). The class subnet (c) is for classifying anchor boxes, and the box subnet (d) is for regressing from anchor boxes to ground-truth object boxes. (Lin, Tsung-Yi, et al., 2017 [32].)

RetinaNet is composed of a backbone network and two subnetworks. Fig 1 shows the overall architecture of RetinaNet. The backbone network computes convolutional feature maps of an entire input image. The first subnetwork is the class subnet which classifies the output of the backbone network and the second subnetwork is the box subnet that performs convolutional bounding box regression. The architecture of RetinaNet is simpler than that of a two-stage object detector that is composed of independent multiple networks for classification and regression.

**Backbone Nnetwork**: The Feature Pyramid Network (FPN) [34] is built on top of the standard CNN ResNet [35] and is the backbone network. FPN generates a rich and multi-scale convolutional feature pyramid by augmenting ResNet with a top-down pathway and lateral connections. The pyramid has 5 levels (P3, …, P7) with 256 channels. Each level of the pyramid can be used for object detection at a different scale.

**Anchor**: Nine translation-invariant anchors, each of a different-size, are used at each pyramid level. A K-class length of one-hot vector of classification targets and a 4-dimensional vector of box regression targets are assigned to each anchor. The assignment rule from the region proposal network (RPN) of Faster R-CNN [28] was used.

**Class subnet**: The class subnet is a small Fully Convolutional Network (FCN) attached to each level of the FPN. The class subnet estimates the probability of object presence at each spatial position for the 9 anchors and K object classes. Four 3 × 3 convolution layers with 256 channels each and ReLU activation, and an additional 3 × 3 convolution layer with 9 × K filters are applied to feature maps from each pyramid level. Finally, sigmoid activation function is applied to the outputs. (see Fig 1(c)).

**Box subnet**: The box subnet which is almost identical to the class subnet is attached to each pyramid level. The only difference is that the box subnet generates 4 × 9 linear outputs per spatial location (Fig 1(d)). For each of the anchors per spatial location, the box subnet computes regressions of the existing offset between a nearby ground-truth box and the anchor box. The box subnet has a network structure similar to that of the object class subnet, but does not share the same parameters with it.

**Focal loss**: Focal loss (FL) was proposed to address the extreme class imbalance between foreground and background when a one-stage object detector is trained. Focal Loss is a simple extension of cross entropy (CE) loss function. CE loss function is defined as Eq (2) when the estimated probability for binary classification is defined as Eq (1).

$$\begin{array}{c} p_{t}=\{\begin{array}{cc} p, & \text{if}\;\text{y}=1\\ 1-p, & \text{otherwise}. \end{array} \end{array}$$ (1)

$$\begin{array}{c} \text{CE}\left(p_{t}\right)=-\text{log}\left(p_{t}\right) \end{array}$$ (2)

$$\begin{array}{c} \text{FL}\left(p_{t}\right)=-\alpha {\left(1-p_{t}\right)}^{\gamma }\text{log}\left(p_{t}\right) \end{array}$$ (3)

The main property of CE loss function is that even samples that are easy to classify have a considerable amount of loss. Using CE loss function guarantees successful result when training a model on a balanced set. However, it is unsuitable for one-stage object detectors including RetinaNet which intentionally generates numerous samples of background to train themselves which can distinguish actual objects and background. For one-stage object detectors, when calculating loss over all generated samples and actual objects, loss from the easy common samples of background can be greater than loss from difficult uncommon samples which are for actual objects. In other words, most of the aggregated loss comes from the common samples for background, which are less important. To address this problem, in Focal Loss function, a modulating factor *α*(1 − *p*_*t*_)^*γ*^ is applied to the CE loss function as defined in Eq (3). The *α* is the weight assigned to the minority class and the parameter *γ* controls the strength of the modulating term. With this modulating factor, one-stage object detectors can get all different loss from each sample and more concentrate on difficult samples during training. The performance enhancement achieved by RetinaNet is mainly attributed to Focal Loss function [32].

### Training and inference procedure

RetinaNet classified objects which are detected into 80 object classes for the object detection task of the COCO challenge [36]. In the mass detection task, however, it needs to classify objects into only the following two binary classes: mass and background. Therefore, the parameter K, which determines the number of classes, for the subnet is set to 2. We use ResNet50 pre-trained on the ImageNet dataset for the backbone network and the Adam optimizer with the learning rate of 0.00001. The *α* is set to 0.25 and the *γ* is set to 2 for the focal loss which were the best values obtained in the original paper [32]. We conduct all the experiments using a single machine with the following configuration: Intel(R) Core(TM) i7-6700 3.30GHz CPU with NVIDIA GeForce GTX 1070 Ti 8GB GPU and 48GB RAM. We reference the Keras implementation of RetinaNet (https://github.com/fizyr/kerasretinanet). The codes for our mass detection model are available at the GitHub repository (https://github.com/hwejin23/MAMMO_Retinanet) (also available in S1 Code).

Due to the extremely small size of the datasets INbreast and GURO (less than 1% of data of general image classification tasks), we use several methods to resolve the data shortage problem. First, as a deep CNN with randomly initialized weights obtains poor performance on small datasets in general, ResNet of the backbone network is pre-trained on the ImageNet dataset [37]. Second, we employ several data augmentation methods to increase the size of the training sets. Single mammograms are divided into small sections based on the location of lesions. All the sections and full mammograms were flipped, randomly cropped, and rotated up to 90°, 180°, and 270° to enlarge the training set.

**Fig 2:**
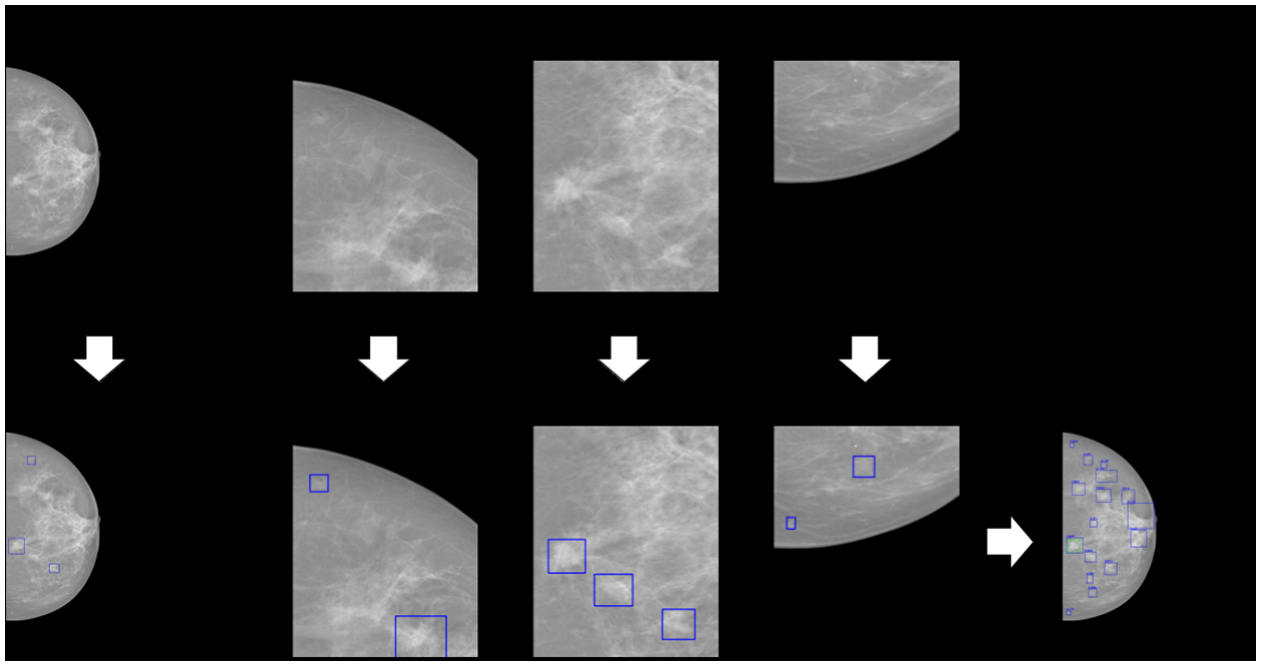
The full mammogram and 25 overlapped sections are used together as an input to our mass detection model. The inference results of each input image are put together.

The size of a single mammogram (around 4000 × 3000 pixels) is too large to use as an input for RetinaNet when conducting inference. We resize the short side of a single mammogram to 600 pixels while keeping the overall ratio. Due to the resizing method, small masses and their original shape can become too small to see. To address this, we divide original mammograms into small sub-sections which do not require the resizing method. To avoid cutting out a part of a mass when dividing mammograms, we divide them into overlapping sections such that half of a section overlaps with an adjacent section. The resized single mammogram and the 25 overlapping sections are used together as an input to our mass detection model for a single inference. The number of bounding boxes per inference for each section is limited to 300. To combine inference results, overlapping bounding boxes are merged when the Intersection over Union (IoU) between two boxes exceeds 0.2. The largest bounding box and the highest confidence score among the set of overlapping inference results are used as the representative bounding box and confidence score, respectively. Additionally, the predicted bounding boxes of areas less than 5000 pixels are excluded from the final mass candidates. The pipeline for inference is presented in Fig 2.

### Transfer learning

Transfer learning is a machine learning technique used to leverage a model trained on one task for another related task [38]. Training the model with random weight initialization on an insufficient dataset does not guarantee successful results. Therefore, researchers who cannot obtain sufficient datasets usually employ another sufficient datasets which are collected for similar task. They train their model on a similar dataset which is sufficient on their own or use publicly available weights pre-trained by other researchers. Using weights pre-trained on datasets that are highly related to their task, researchers fine-tune the model on the actual dataset they wish to use. In the medical image analysis domain, transfer learning can be used to obtain accurate annotations of lesions since it is difficult to collect a sufficient number of well refined images due to privacy issues. Therefore, we study the effectiveness of transfer learning through experiments using public and in-house mammogram datasets, and make the weights pre-trained on our datasets publicly available so that other researchers in the mammography community can leverage them.

## Experiments and results

### Data

The experiments were conducted on the public dataset INbreast [33] and the in-house dataset GURO. INbreast was obtained from the S. João Hospital Centre in Porto. It consists of 410 full digital mammograms from 115 patients, which were annotated with coordinates of 116 masses. All lesions including masses were assigned a standardized Breast Imaging-Reporting and Data System (BI-RADS) category [39] by a radiologist after interpreting a mammogram. Since the malignancy labels were not validated by biopsy, we use all the lesions labeled as masses regardless of malignancy.

GURO is an in-house dataset developed by Korea University Guro Hospital. It contains full digital mammograms, taken by various screening systems, of 350 breast cancer patients who were treated by one of the co-authors in this study. All the mammograms were collected with the approval of the Institutional Review Board (IRB) of Korea University Guro Hospital (Approval number: KUGH15342-006). Of the 350 patients, 111 patients have masses. Therefore, 222 full digital mammograms of 111 patients are used. Each image contains a mass and the biopsy proves that all the masses are malignant. The locations of the masses were manually annotated as bounding boxes by experienced radiologists of Korea University Guro Hospital. These annotations were used as the ground truth labels.

In INbreast, the borders of the masses are annotated using a number of points. Since our mass detection model uses bounding boxes as input, provided annotations should be converted into bounding boxes. Therefore, the bounding boxes are created based on minimum and maximum points of x-coordinates and y-coordinates, which indicate the locations and shapes of masses. We found that our mass detection model generally fails to capture appearance features when it obtains a bounding box that contains only mass with almost no background. So, we enlarge the size of the bounding boxes by increasing the widths and heights by 10%. This tactic allows the annotated bounding boxes to contain more backgrounds so that the masses can be more easily distinguished from the background.

### Experimental setups

Our seven experimental setups, denoted as S-1, S-2, and so on, for evaluation are shown in Table 1. In S-1, INbreast was used for training and testing, and in S-2, GURO was used for training and testing. According to the research, the breasts of Asian women are relatively denser than those of Western women [40]. Thus, we study how the variation in patient profiles affects the mass detection results as the mammograms of INbreast and GURO were collected from patients of different races. In S-3, both GURO and INbreast were combined and used as the training set and only INbreast was used as the test set. In S-4, GURO and INbreast were combined and used for training, and GURO for testing. Finally, both INbreast and GURO were combined and divided into the training and test sets in S-5. We conduct supplementary experiments for S-1 and S-2 to examine the effect of transfer learning. In S-6, GURO was used for pre-training, and INbreast was used for training and testing. On the other hand, in S-7, INbreast was used for pre-training, and GURO was used for training and testing. We performed a patient-wise five-fold cross-validation in all the experimental setups. 80% and 20% of all the mammograms from each setup are randomly selected and used as the training set and the test set, respectively. For a fair comparison, the test sets of S-1, S-3, S-6 contain the same mammograms as INbreast and the test sets of S-2, S-4, S-7 contain the same mammograms as GURO. In all the training and testing phases, the mammograms in the training set are strictly excluded from the test set.

**Table 1:** Experimental setups.

Setup Name	Pre-trained on	Training Set	Test Set
S-1	-	INbreast	INbreast
S-2	-	GURO	GURO
S-3	-	INbreast + GURO	INbreast
S-4	-	INbreast + GURO	GURO
S-5	-	INbreast + GURO	INbreast + GURO
S-6	GURO	INbreast	INbreast
S-7	INbreast	GURO	GURO

### Results

**Fig 3:**
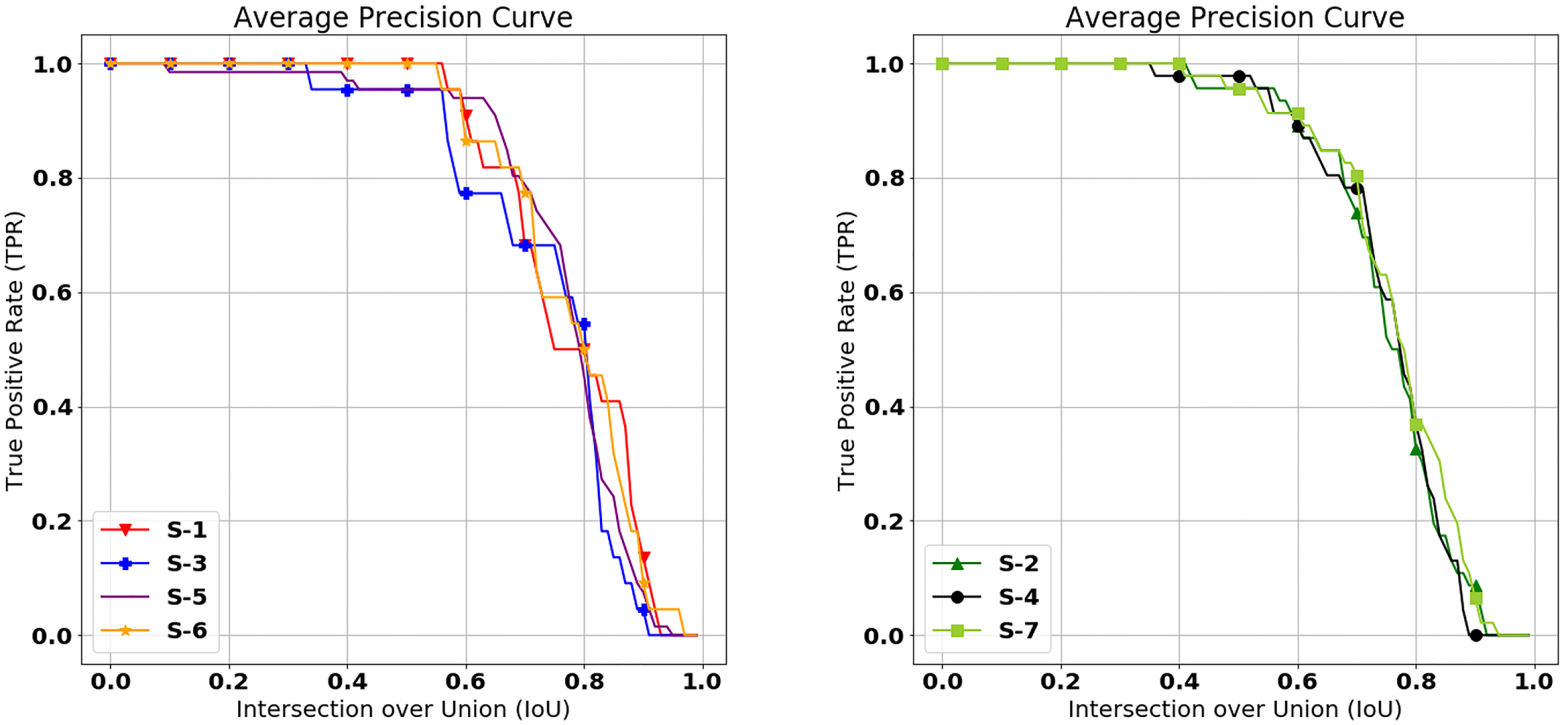
True positive rate as a function of the minimum IoU value. The left figure shows the average precision curves of S-1, S-3, S-5, and S-6 which are results of setups tested on INbreast and the right figure shows the average precision curves of S-2, S-4, and S-6 which are the results of setups tested on GURO.

Fig 3 shows the true positive rate (TPR) at different minimum IoU values between ground truth and inference results. Although the results of each experimental setup differ, the FROC curves of all the results have a similar shape. In S-1, which uses only INbreast, the true positive rate remains stable when the IoU value is less than 0.55, and it starts to fall when the IoU value is greater than 0.55. This result demonstrates that our mass detection model accurately detects masses since the IoU value of 0.55, which allows stable high performance, is larger than IoU value of 0.2, which is commonly used IoU value for conventional mass detection models [4, 12, 13, 15].

**Fig 4:**
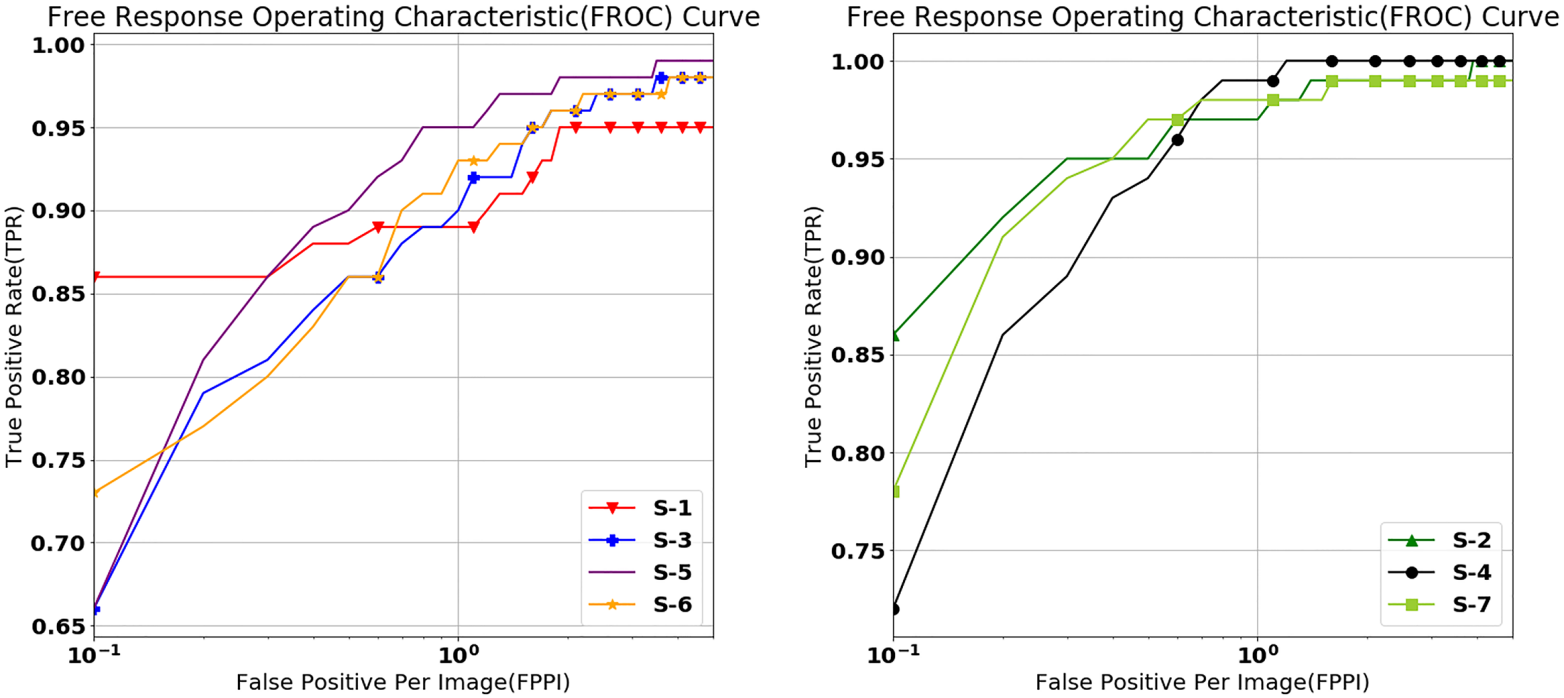
Free response operating characteristic (FROC) curves of the results on various operating points, representing the true positive rate (TPR) and the false positive per image (FPPI). FPPI values on the X-axis are converted to a logarithmic scale. The left figure shows the FROC curves of S-1, S-3, S-5, and S-6, which denote the results on INbreast, and the right figure shows the FROC curves of S-2, S-4, and S-6, which denote the results on GURO.

We use the free response operating characteristic (FROC) curves for evaluating the performance of our mass detection model. The FROC curves show the true positive rate (TPR) and the false positives per image (FPPI). The FROC curves obtained by five-fold cross validation for all the experimental setups are shown in Fig 4. The IoU value for accepting the inference result as the correct answer is set to 0.2. As the FROC curves show in Fig 4, our mass detection model yields a TPR of 0.95 ± 0.04 for S-1, 0.99 ± 0.02 for S-2, 0.97 ± 0.02 for S-3, 0.99 ± 0.01 for S-4, 0.97 ± 0.02 for S-5, 0.97 ± 0.02 for S-6, and 0.99 ± 0.02 for S-7 with a FPPI = 3. Similarly, our mass detection model yields a TPR of 0.91 ± 0.07 for S-1, 0.98 ± 0.02 for S-2, 0.92 ± 0.05 for S-3, 1.0 ± 0.0 for S-4, 0.98 ± 0.02 for S-5, 0.94 ± 0.05 S-6, and 0.98 ± 0.02 for S-7 with a FPPI = 1.3, which suggests that our mass detection model achieves high accuracy with low false positives.

S-3 and S-4 use the combined training set of INbreast and GURO. Performance is retained and slightly improved in S-3 and S-4, respectively, both of which use only single training sets. Also, performance achieved in S-5, which uses the combined INbreast and GURO set for training and testing, is comparable to that of other setups. The results indicate that using more training data even though they are obtained from different sources can further enhance performance. Moreover, the results demonstrate that our mass detection model can potentially further improve performance in actual diagnosis after deployment as more data will be generated over time.

The performance in experimental setups S-6 and S-7, both of which employ weights pre-trained on GURO and INbreast, is comparable to that achieved in S-3 and S-4, respectively, where additional datasets are not used for pre-training a model but used directly in training. These results suggest that weights pre-trained on a dataset can be used to leverage the features learned from the dataset. We are unable to disclose the in-house dataset GURO due to patient privacy issue. As an alternative, rather than the dataset itself, we release our weights pre-trained on INbreast and GURO, which can be used to obtain the similar results as directly using the dataset. Our weights pre-trained on INbreast and GURO are available at the GitHub repository (https://github.com/hwejin23/MAMMO_Retinanet).

**Fig 5:**
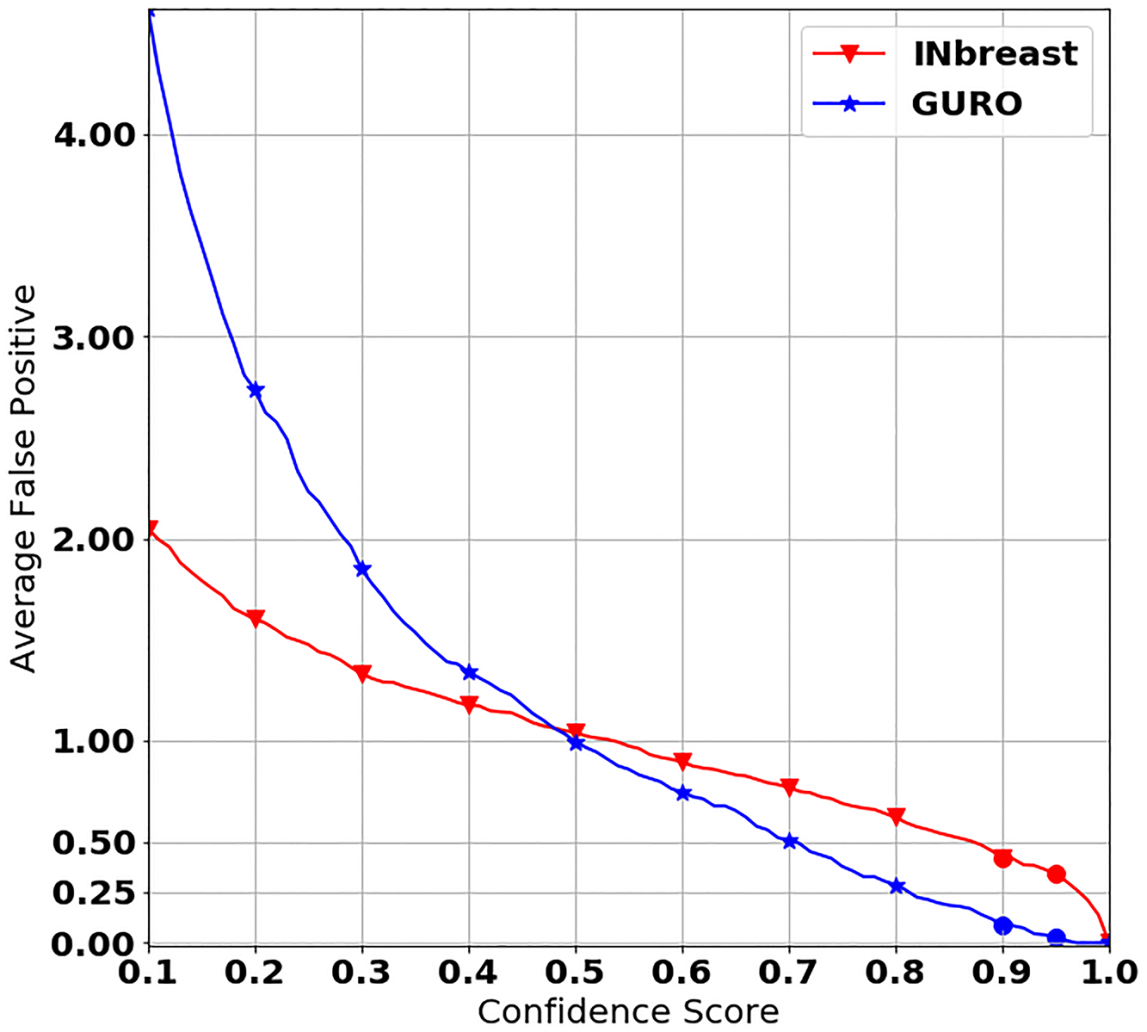
X of the curve is the average number of false positives per image, and Y of the curve is the confidence score of our mass detection model. To show how well our mass detection model works on mammograms without masses, we trained our model on the mammograms containing masses and tested it on the mammograms without masses. The red line denotes the result on INbreast and the blue line denotes the result on GURO. To make it easier to distinguish between similar values, we converted the scale to a log scale, and the confidence score starts at 0.1.

In terms of mass detection performance, it is also important to consider the number of false positives generated by our mass detection model used on mammograms without masses. To validate performance on mammograms without masses, we trained our mass detection model on all mammograms with masses and tested our model on mammograms without masses. Fig 5 shows the results from the experiments that use mammograms without masses. This figure represents the average number of false positives per image on the Y-axis and the confidence score on the X-axis. The result of INbreast is denoted by a red line and the result of GURO is represented by a blue line. As shown in Fig 5, as the confidence score increases, the average number of false positives decreases for both datasets. In INbreast, the average number of false positives is 0.42 when the confidence score is 0.9, and it decreases to 0.34 when the confidence score is 0.95. In GURO, the average number of false positives is 0.09 when the confidence score is 0.9 and it decreases to 0.03 when the confidence score is 0.95.

Table 2 compares our experimental results of all the setups with the results of the state-of-the-art mass detection models. Although it is difficult to make a direct comparison because each of the studies had somewhat different settings and datasets, an overall performance comparison is possible. Our mass detection model achieves high TPR value comparable to that of the recent deep learning-based state-of-the-art mass detection models, which is significant because our model is a simple end-to-end one-stage object detector. It also yields an overwhelmingly higher TPR than conventional models that use machine learning techniques with hand-crafted features. Moreover, our mass detection model shows the fastest inference time of 1.8s per mammogram.

**Table 2:** Performance comparison of the mass detection models.

Paper	Method	Dataset (Testing on)	TPR@FPPI	Inference time (Machine spec.)
Ours	Deep Learning	S-1 (INbreast)	0.95 ± 0.04 @ 3.0	1.8s (Intel Core i7, NVIDIA GTX 1070)
			0.91 ± 0.07 @ 1.3	
			0.88 ± 0.07 @ 0.5	
		S-2 (GURO)	0.99 ± 0.02 @ 3.0	
			0.98 ± 0.02 @ 1.3	
			0.95 ± 0.02 @ 0.5	
		S-3 (INbreast)	0.97 ± 0.02 @ 3.0	
			0.92 ± 0.05 @ 1.3	
			0.86 ± 0.06 @ 0.5	
		S-4 (GURO)	0.99 ± 0.01 @ 3.0	
			1.00 ± 0.00 @ 1.3	
			0.94 ± 0.03 @ 0.5	
		S-5 (Combinded set)	0.97 ± 0.02 @ 3.0	
			0.98 ± 0.02 @ 1.3	
			0.90 ± 0.04 @ 0.5	
		S-6 (INbreast)	0.97 ± 0.02 @ 3.0	
			0.94 ± 0.05 @ 1.3	
			0.86 ± 0.03 @ 0.5	
		S-7 (GURO)	0.99 ± 0.02 @ 3.0	
			0.98 ± 0.02 @ 1.3	
			0.97 ± 0.01 @ 0.5	
Ribli et al. [24]	Deep Learning	INbreast*	0.90 @ 0.3	N/A
Choukroun et al. [25]	Deep Learning	IMG	0.76 @ 0.48	N/A
Akselrod-Ballin et al. [23]	Deep Learning	INbreast	0.93 @ 0.56	5s (Intel Core i7, NVIDIA TitanX)
		internal	0.9 @ 1	
Dhungel et al. [21]	Deep Learning	INbreast	0.95 ± 0.02 @ 5.0	39s (Intel Core i5, NVIDIA GTX 460)
			0.90 ± 0.02 @ 1.3	
Dhungel et al. [41]	Deep Learning	INbreast	0.90 ± 0.02 @ 1.3	39s (Intel Core i5, NVIDIA GTX 460)
Dhungel et al. [20]	Deep Learning	INbreast	0.96 ± 0.03 @ 1.2	20s (Intel Core i5)
			0.87 ± 0.014 @ 0.8	
		DDSM	0.75 @ 4.8	
			0.70 @ 4.0	
Kozegar et al. [16]	Ensemble Classifier	INbreast	0.87 @ 3.67	108s (Intel Core 2 Duo)
Sampat et al. [15]	Rule Based Method	DDSM	0.88 @ 2.7	N/A
			0.85 @ 1.5	
			0.80 @ 1.0	
Eltonsy et al. [14]	Rule Based Method	DDSM	0.92 @ 5.4	N/A
			0.88 @ 2.4	
			0.81 @ 0.6	
Bellotti et al. [13]	Neural Network	MAGIC-5	0.80 @ 4.23	N/A
Beller et al. [12]	Decision Tree	DDSM	0.70 @ 8.0	N/A
Wei et al. [11]	Linear Discriminant Analysis	University of Michigan	0.90 @ 2.0	N/A
			0.80 @ 1.2	
			0.70 @ 0.79	
Campanini et al. [10]	SVM	DDSM	0.80 @ 1.1	N/A
te Brake et al. [9]	Nerual Network	Nijmegen	0.70 @ 0.1	N/A

*Note1*: INbreast* is a reconstructed dataset whose lesions are manually selected within INbreast.

**Fig 6:**
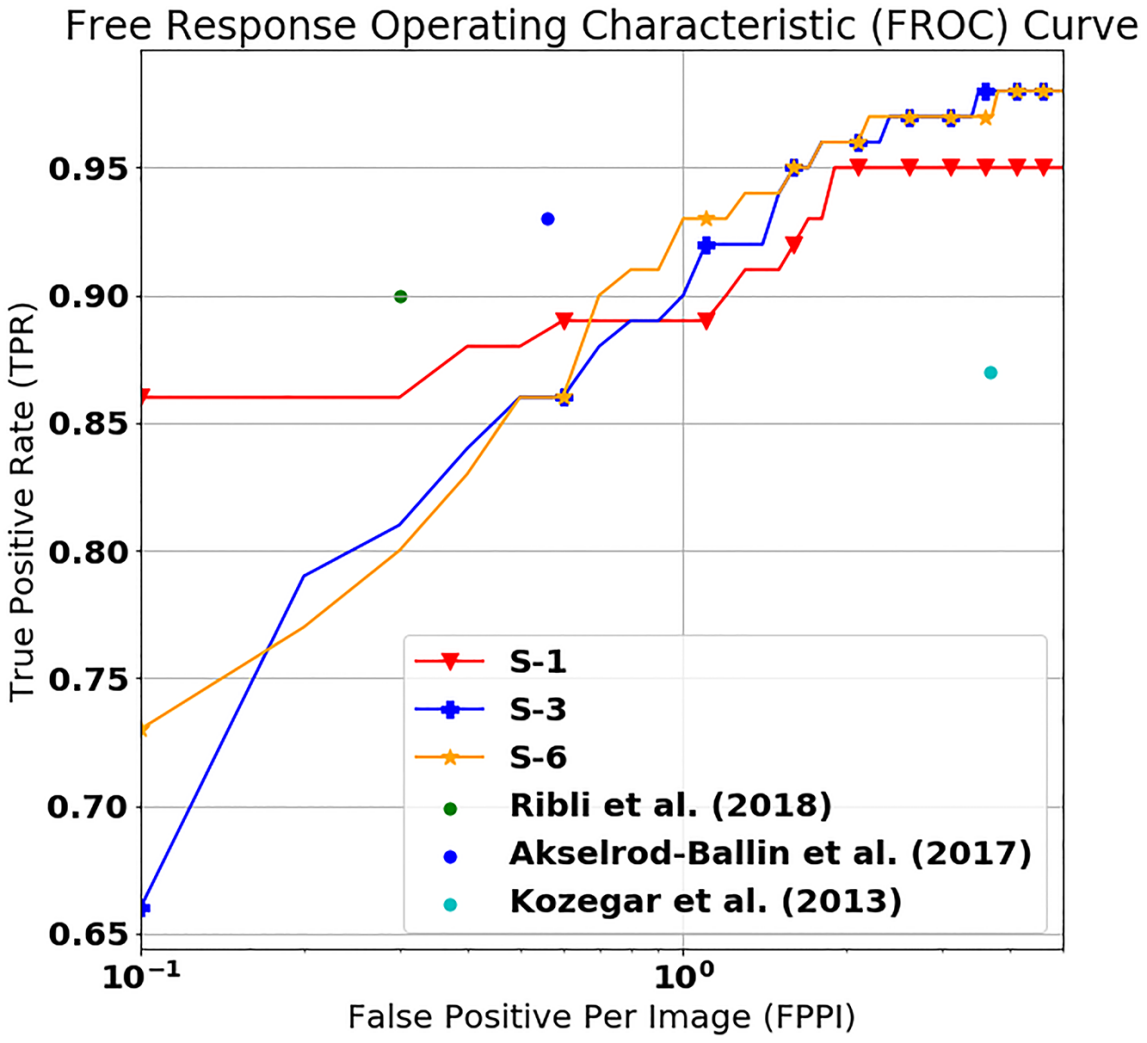
FROC curves of our mass detection model used in S-1, S-3, and S-6 and the announced results of state-of-the-art mass detection models. FPPI values on the X-axis are converted to a logarithmic scale.

The performance comparison between our mass detection model and the other state-of-the-art models presented in Fig 6. For a fair comparison, we excluded the experimental results tested on the in-house dataset and the Digital Database for Screening Mammography (DDSM) [42] which is a film-based mammogram dataset. The FROC curves of our mass detection model used in S-1 (training and testing on INbreast), S-3 (training on INbreast and GURO, testing on INbreast), and S-6 (pre-training on GURO, training and testing on INbreast) are indicated by the red, blue, and orange lines, respectively. We plotted the results of three mass detection models developed by Ribli et al. (2018) [24], Akselrod-Ballin et al. (2017) [23], and Kozegar et al. (2013) [16], respectively. The models of Ribli et al. and Akselrod-Ballin et al. outperformed our model. We believe this is attributed to the size and composition of the dataset. The lesion detection model of Ribli et al. was trained on mammograms containing not only masses but also calcifications, and thus the performance of their model was evaluated on different types, and not only on masses. Moreover, their training and test sets for the experiment were composed of lesions manually selected and considered as malignant by the authors. Therefore, a direct comparison of the results of Ribli et al. and ours is difficult. The mass detection model of Akselrod-Ballin et al. was trained on their in-house dataset which contains 750 mammograms each of which has a mass. Less than 300 mammograms were used as the training set in our experiments (S-1, S-3, S-6). Our results are competitive with those of current state-of-the-art deep learning-based mass detection models.

**Fig 7:**
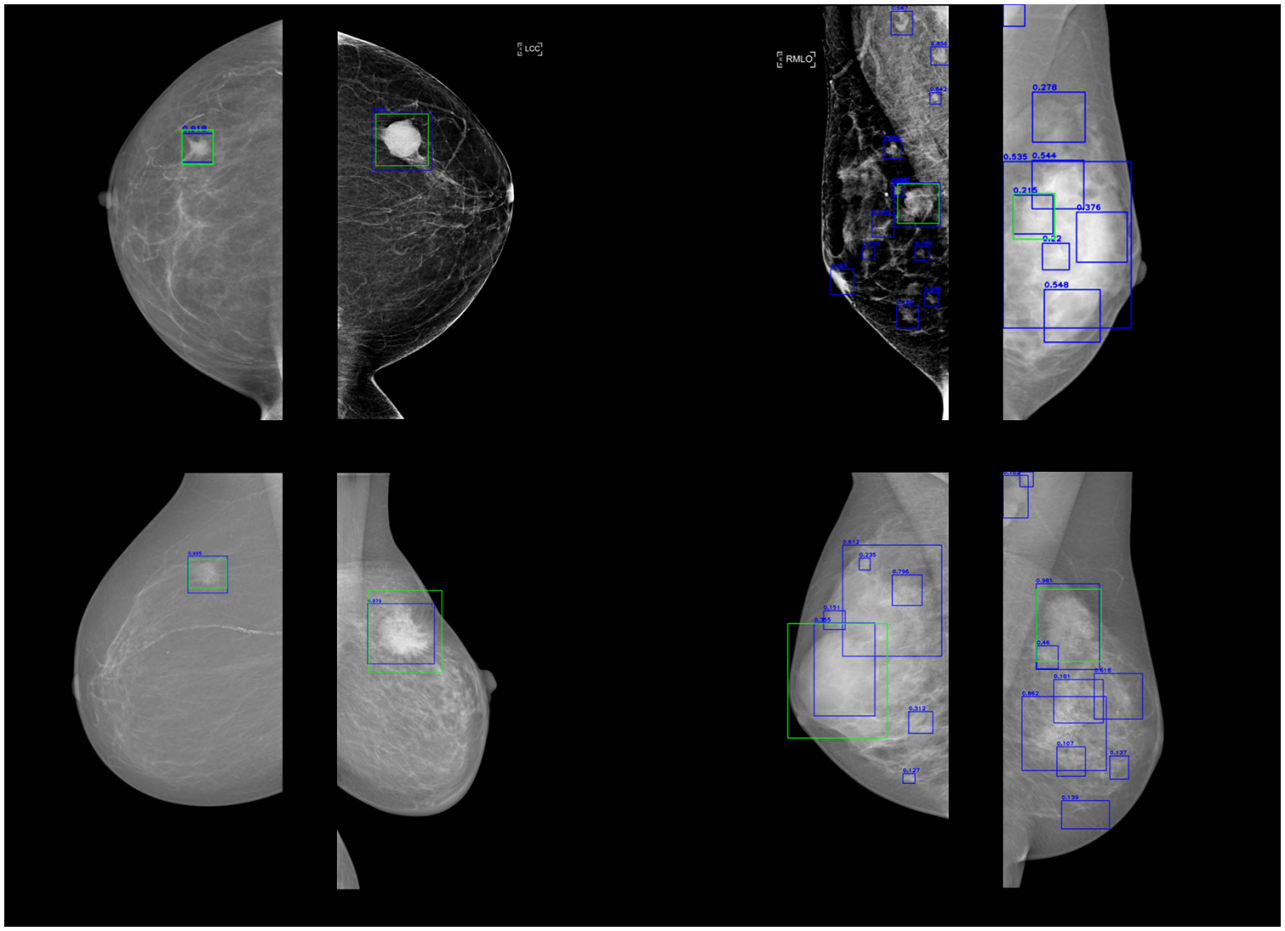
Several good and poor cases from the S-1 and S-2 setups. Mammograms from GURO are shown at the top and mammograms from INbreast are shown at the bottom.

Several prediction results are shown in Fig 7. The top row shows mammograms from GURO and the bottom row shows mammograms from INbreast. Ground truth annotations are displayed in green and the predicted boxes with their confidence scores from our mass detection model are outlined in blue. Fig 7(a), 7(b), 7(e) and 7(f) show mammograms that have a low number of false positives. A single mass detection result at the exact position with a high confidence score is predicted for each mammogram. However, Fig 7(c), 7(d), 7(g) and 7(h) show mammograms that have an excessive number of false positives. Nodules in pectoral muscles, which look similar to masses, are also misidentified as masses in each mammogram. Mammograms of the patients who have dense breasts are likely to have this kind of error. It is difficult even for experienced radiologists to detect masses in such poor cases. The shape of the mass in Fig 7(g) (INbreast) is unusual and uncommon. Masses are typically highly dense, small, and round, but the mass in Fig 7(g) is not round and is large as the breast. An insufficient number of abnormal masses causes a limitation for our mass detection model.

## Discussion

Our mass detection model demonstrates state-of-the-art performance. However, performance can be further improved by addressing the issues identified below.

**Mass malignancy**: INbreast contains both benign and malignant masses. Although the BI-RADS categories can help determine whether a lesion is malignant, an actual biopsy for diagnosis is not conducted; thus, using the BI-RADS categories cannot be considered as an accurate method for determining malignancy. On the other hand, all the masses from GURO are diagnosed as malignant based on the biopsy results. Therefore, if a clear difference in appearance exists between the benign and malignant masses, it can affect the performance of deep convolutional neural network based models.

**Data shortage**: The amount of data used in our study is insufficient for training deep networks. A small amount of training data cannot represent all types of masses and can lead to over-fitting. Data augmentation and fine-tuning techniques with pre-trained weights were applied to address the lack of data. While these methods could enhance performance, fundamental improvement in model performance can be achieved only with a larger training set that contains a sufficient number of diverse cases.

## Conclusion

In this paper, we introduced a mass detection model based on RetinaNet which is a state-of-the-art one-stage object detector. We evaluated our mass detection model in various experimental setups with the public and in-house datasets. Our mass detection model achieved a true positive rate similar to that of the state-of-the-art mass detection models, and outperformed the conventional mass detection models, which proves its effectiveness. The overall performance was retained or slightly improved when larger integrated datasets were used for training even though they were obtained from different sources. This result suggests that the performance of our mass detection model can be further improved by training on large size in-house datasets of other researchers and medical institutions. Furthermore, we also validated the performance of our mass detection model in the transfer learning experiment. We made our weights that were pre-trained on the in-house dataset GURO and the public dataset INbreast available for community use.

## Supporting information

S1 Code Python source code. All code used in this study is available at: https://github.com/hwejin23/MAMMO_Retinanet.
(ZIP)
